# Metformin Actions on the Liver: Protection Mechanisms Emerging in Hepatocytes and Immune Cells against NASH-Related HCC

**DOI:** 10.3390/ijms22095016

**Published:** 2021-05-09

**Authors:** Yueqi Zhang, Hongbing Wang, Hua Xiao

**Affiliations:** 1 Cell and Molecular Biology Program, College of Natural Science, Michigan State University, East Lansing, MI 48824, USA; zhang878@msu.edu; 2 Department of Physiology, College of Natural Science, Michigan State University, East Lansing, MI 48824, USA; wangho@msu.edu

**Keywords:** metformin, NAFLD, NASH, HCC, type 2 diabetes mellitus, macrophage, T cell, myeloid-derived suppressor cell, MDSC

## Abstract

Nonalcoholic fatty liver disease (NAFLD) is strongly linked to the global epidemic of obesity and type 2 diabetes mellitus (T2DM). Notably, NAFLD can progress from the mildest form of simple steatosis to nonalcoholic steatohepatitis (NASH) that increases the risk for hepatocellular carcinoma (HCC), which is a malignancy with a dismal prognosis and rising incidence in the United States and other developed counties, possibly due to the epidemic of NAFLD. Metformin, the first-line drug for T2DM, has been suggested to reduce risks for several types of cancers including HCC and protect against NASH-related HCC, as revealed by epidemical studies on humans and preclinical studies on animal models. This review focuses on the pathogenesis of NASH-related HCC and the mechanisms by which metformin inhibits the initiation and progression of NASH-related HCC. Since the functional role of immune cells in liver homeostasis and pathogenesis is increasingly appreciated in developing anti-cancer therapies on liver malignancies, we discuss both the traditional targets of metformin in hepatocytes and the recently defined effects of metformin on immune cells.

## 1. Introduction

The global epidemic of obesity and type 2 diabetes mellitus (T2DM) lowers individuals’ quality of life and lifespan and increases the risk of life-threatening comorbidities, including various types of cancers. People with obesity or T2DM often have excessive lipid storage in the liver, which is a marking characteristic of nonalcoholic fatty liver disease (NAFLD). Insulin resistance, which is a pathological condition linked to prediabetes and T2DM, also results in nonalcoholic fatty liver (NAFL) spontaneously. Thus, patients with T2DM would inevitably develop some forms of NAFLD. When NAFLD progresses and causes hepatic inflammation and hepatocyte damage, it is known as nonalcoholic steatohepatitis (NASH). While simple steatosis or NAFL is not regarded as a significant risk factor for adverse outcomes in patients [1], NASH increases the risk of liver failure and hepatocellular carcinoma (HCC) [2], which is a primary liver cancer with a dismal prognosis and few therapeutic options in the advanced stage. With cell death and chronic inflammation in NASH, fibrosis usually develops and can progress to cirrhosis, a well-established risk factor for HCC [3]. It should be noted that NASH-related HCC can develop in the absence of cirrhosis or fibrosis [4]. In the US, while hepatitis viral infection is still a major etiology for HCC, the metabolic syndrome is recognized as the emerging driving force for HCC incidence growth. Along with the escalating prevalence of obesity, HCC has become the fastest growing cancer in the US [5]. Moreover, metabolic disorder-related HCC was observed to have higher mortality risk as compared to other non-alcoholic etiologies including HBV and HCV [6]. The increasing prevalence of NAFLD, which is also known as the hepatic manifestation of the metabolic syndrome, may directly account for the growth of HCC incidence and mortality [5].

Metformin is the first-line drug in most clinical practice guidelines for T2DM with low cost, robust efficacy, and good tolerability. While the mechanism of action is still not fully understood, metformin has been known to lower the overall glucose level by primarily acting on the liver, where it can reduce gluconeogenesis and glycogenolysis and also improve several features of NAFLD. The anti-cancer efficacy of metformin is suggested by several studies where an intriguing association of metformin use with reduced cancer mortality and incidence in T2DM patients was observed [7,8]. Mechanistic studies and ongoing clinical trials also demonstrated the anti-neoplastic effects of metformin [9,10,11]. The possible preventive effect of metformin against HCC may not be exclusively mediated by lowering glucose and insulin levels since its efficacy against HCC is superior to that of other anti-diabetes medications [12,13,14].

Lines of evidence demonstrate that the immune system is functionally involved in the development of hepatic insulin resistance and steatosis as well as the initiation and progression of cancers, including HCC. However, it is unclear whether the immune system impinges on these distinct but potentially connected pathological outcomes through a common mechanism. Intriguingly, the effects of metformin on insulin resistance and NASH are concurrent with its impacts on inflammation and immune cell function. Given that inflammation is a key precondition for HCC development, metformin may have a novel value in preventing and treating NASH-related HCC by regulating the immune system.

Currently, both the effect and mechanism of metformin in preventing or treating HCC are not fully understood. In this review, we summarize the recent evidence on reducing the risk and progression of NASH-related HCC by metformin. We discuss the critical events in the pathogenesis and progression of NASH-related HCC. We explore and discuss the activities of metformin against these key events either in the hepatocyte or in the immune cells, including macrophages, myeloid-derived suppressor cells (MDSCs), and various T cells.

## 2. Reduced Risk and Progression of HCC in NAFLD/NASH Patients by Metformin

Metformin as a treatment for NAFLD/NASH has been extensively examined by pilot studies and randomized controlled clinical trials. The results were mixed. While metformin benefits liver functions, as indicated by the decreased alanine aminotransferase level and the improved insulin sensitivity, it shows little effect on liver histology. As a result, metformin is not recommended by the American Association for the Study of Liver Diseases as a treatment for NASH [15]. For the time being, there is no trial testing the protective effect of metformin for HCC in NAFLD/NASH patients.

Since metformin is the first-line drug for T2DM and up to two-thirds of T2DM patients have NAFLD [15,16], retrospective observational studies may provide helpful information regarding the association between metformin use and the incidence of NASH-related HCC. In a cohort study of 100 diabetic patients with HCV cirrhosis, metformin use significantly associates with a reduced HCC incidence and a lower requirement rate for liver transplantation or the rate of liver-related death [17]. Of the 91 patients who had liver biopsies, more than 52% of patients had macro-vesicular steatosis affecting at least 5% of hepatocytes, indicating that they might be diagnosed with NAFLD [17,18]. Kasmari et al. reviewed the US insurance database. They found that, while excluding individuals with concomitant cirrhosis, NAFLD, and NASH, the HCC incidence in T2DM patients who received metformin treatment was still significantly lower [19]. Analysis of Taiwan’s National Health insurance database also found that metformin use was significantly associated with reduced HCC incidence. Notably, such a reduction was seen in all patient subgroups with and without liver-related diseases. NAFLD/NASH might be included in the “other chronic nonalcoholic liver disease” category [20]. In a cohort of 191 T2DM patients in the US with histologically confirmed NASH and fibrosis, metformin use was significantly associated with lower HCC risk and a reduced all-cause mortality rate and transplantation rate [21]. In an international multicenter study, clinical data from a cohort of 299 patients from different continents with biopsy-proven NASH and cirrhosis were analyzed for the association of clinical factors, including the use of metformin, with patient outcomes. Vilar-Gomez et al. found that metformin use was associated with a higher probability of transplant-free survival as compared to non-users. In addition, continuous metformin use was associated with a reduced risk of HCC and all-cause mortality [22]. Thus, there is evidence that metformin use may reduce the incidence and death rate of NASH-related HCC, albeit, such a reduction is not limited to NASH-related HCC but also appears in HCC with other etiologies. With respect to directly evaluating metformin as a treatment option for HCC, several ongoing clinical trials combine metformin with PD-1 antibodies or others for treating HCC. To date, no complete result is available.

Even though using metformin as a treatment for NAFLD/NASH is not recommended at this time, several clinical studies have examined the association of metformin use with HCC risk or mortality in NASH patients. A significant association was found. More such studies are urgently needed in the current situation as the NAFLD burden is ramping up, and NASH is becoming the main force driving HCC incidence.

## 3. Links between NASH and HCC

**Figure 1:**
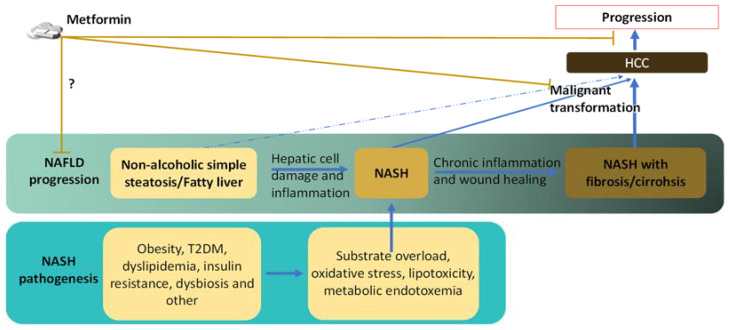
Pathogenesis of NASH and NASH-related HCC and the possible effect of metformin on these processes. NAFLD can progress from the mildest form of simple steatosis to NASH, which is defined by hepatic cell damage and inflammation that are absent in the simple steatosis stage. The current theory acknowledges the development of NASH as a result of “multiple hits” that overload and stress the hepatocyte. The chronic inflammation, cell damage, and wound healing processes in the NASH condition cause fibrosis and cirrhosis. While simple steatosis is not a major risk factor for HCC, NASH conditions, especially those with cirrhosis, increase HCC risk. Although there is disagreement regarding whether metformin can inhibit NAFLD progression, preclinical and clinical studies have provided evidence that metformin use can reduce the risk of NAFLD/NASH-related HCC and suppress HCC progression.

Elucidating the pathogenesis and manifestation of NASH provides essential information to identify HCC initiation mechanisms in the NASH condition and understand how metformin works against it. Chronic liver disease is a critical precondition for HCC development in adulthood. The malignant transformation of the hepatocyte is widely recognized as the result of accumulated genetic and epigenetic alterations from the long-term, vicious cycle of hepatocyte damage, inflammation, and compensatory proliferation of hepatocytes. Thus, both the risk factors that induce NASH and the pathological abnormalities of established NASH that promote the vicious cycle and alterations could contribute to the initiation of HCC over time (Figure 1).

### 3.1. Risk Factors and Preconditions That Link NASH to HCC

The current model for the pathogenesis of NAFLD/NASH is the “multiple-hit” theory [23], where multiple insults disrupt the normal metabolic activity of the liver, resulting in a highly heterogeneous disease in terms of both driving mechanisms and manifestations. The various hits that induce NASH, including many inter-linked factors, such as obesity, T2DM, insulin resistance, dyslipidemia, and altered microbiome, may also promote HCC.

These hits are common in individuals with NAFLD/NASH. A meta-analysis of global NAFLD epidemiology revealed that 81.83% of NASH patients had obesity, and 43.63% of NASH patients had T2DM [2]. Moreover, in a cohort of patients with hypertransaminasemia and T2DM, all patients had biopsy-confirmed NASH [24]. Up to 70% of individuals with obesity have dyslipidemia [25]. Furthermore, as diacylglycerol and ceramide inhibit insulin signaling [26,27], it is suggested that dyslipidemia may directly contribute to insulin resistance in NAFLD. Elevated serum insulin and glucose levels are usually seen in individuals with impaired insulin sensitivity. While the cause-effect relationship among all these conditions and NASH is tangled, these conditions overwhelm the hepatocyte with carbohydrates and fatty acids. The excessive metabolic substrates promote the metabolic abnormality and the production of toxic metabolites in hepatocytes that stress and damage the cell, which further demolishes hepatocytes’ ability to handle substrates [28]. This cycle results in hepatocyte injury and subsequent hepatic inflammation, which separates NASH from simple NAFL and promotes the development of HCC.

Many of these hits inducing NAFLD/NASH cause oxidative stress in the hepatocyte, which may induce oxidative DNA damage over time. Specifically, fatty acid overload in hepatocytes leads to the overproduction of reactive oxygen species (ROS) in NASH. Insulin resistance in adipose tissue induces increased lipolysis and fatty acid flux into the liver. High glucose availability in T2DM, hyperinsulinemia in insulin resistance, and high dietary sugar intake also promote the de novo fatty acid synthesis in hepatocytes [29]. In response to fatty acid overload, there is an increase in fatty acid β-oxidation at the expense of ROS production in the hepatocyte. The fatty acid is also disposed of by conversion into triglycerides, which is then either transported out as VLDL or stored in lipid droplets, that are neutral forms of lipids usually recognized as non-toxic. The excessive fatty acid can also lead to the overproduction of diacylglycerols and ceramides, which can induce lipotoxicity by promoting ROS overproduction and triggering hepatocyte apoptosis [30]. NAFLD patients usually have elevated bile acid levels throughout the body, which links closely to the leaky gut condition and dysbiosis in NAFLD patients [31]. Increased bile acid is hepatotoxic, where it generates ROS and damages the mitochondria [32]. The leaky gut condition, which is related to the ability of bile acid to increase intestinal permeability, enables endotoxins from the microbiome to activate the host immune system and, in turn, causes hepatocyte stress and damage. Oxidative stress, lipotoxicity, and hepatotoxic bile acid also induce endoplasmic reticulum (ER) stress, which is considered the hub of hepatocarcinogenesis. Moreover, chronic low-grade ER stress, similar to that observed in mice fed a high-fat diet, leads to increased hepatic gluconeogenesis and, in turn, results in hyperglycemia, forming another vicious cycle [33]. In summary, several risk factors and preconditions for NAFLD/NASH could induce both oxidative stress and ER stress well known to promote hepatocarcinogenesis.

There are other factors involved in NAFLD/NASH development that may link to HCC development. An emerging view for the causative role of macrophages in the pathogenesis of NAFLD/NASH is that hepatic macrophages can function in this process independently of their pro-inflammatory status. Specifically, macrophages can promote disease establishment by producing non-inflammatory factors, such as IGFBP7 and miR-144, which regulate the antioxidative response and metabolism of hepatocytes [34,35]. Whether dysregulated hepatic macrophages can promote hepatocarcinogenesis with non-inflammatory factors is still an open question, but such a possibility should not be neglected. In addition, hyperinsulinemia usually seen in preconditions of NAFLD/NASH may promote HCC development through several mechanisms. On the one hand, insulin can promote the proliferation of malignant cells by activating the insulin receptor IR-A isoform, which is less common and mainly expressed in fetal and cancer cells. The activation of the IR-A isoform stimulates the activation of ERK1/2 and AKT and, in turn, leads to anti-apoptotic and pro-proliferative effects [36,37]. On the other hand, a high serum level of insulin increases the hepatic expression and surface translocation of the growth hormone receptor (GHR) [38]. This leads to elevated activation of GHR, thereby, stimulating the production and secretion of mitogenic insulin-like growth factor 1 (IGF-1), which has been found to increase in different malignancies, including HCC. Moreover, a high insulin level also reduces the expression of IGF-1 binding proteins, leading to higher IGF-1 bioavailability and, consequently, an elevated activity and mitogenic effect [39]. Other functionally relevant mechanisms that link hyperinsulinemia to HCC include an insulin-mediated increase of matrix protein secretion and impairment of β-oxidation of fatty acids [40].

### 3.2. Links between the Pathological Abnormalities of Established NASH and the Development of HCC

Patients with fully established NASH have a higher risk (annual incidence from 2.4% over seven years to 12.8% over three years, or 5.29 per 1000 person-years) for HCC compared to individuals with obesity or patients with simple NAFLD (0.44 per 1000 person-years) or T2DM [2,41,42,43]. Particular abnormalities of established NASH compared to metabolic syndrome or simple steatosis likely promote HCC initiation. Such abnormalities, including DNA damage, hepatic inflammation and dysregulated immunity, and fibrosis/cirrhosis, are discussed here.

#### 3.2.1. DNA Damage

NASH patients with HCC have more hepatic DNA damages resulting from oxidative stress as compared to NASH patients without HCC [44], which might be caused by various mechanisms. These include elevated oxidative stress, the survival of hepatocytes with excessive DNA damage, changes in the DNA damage repair process, or a combination of these in patients with NASH-related HCC. The first circumstance may result from long-term necroinflammation or stress from chronic dysregulated metabolism in hepatocytes, as discussed earlier. Abnormal consecutive activation of the antioxidative KEAP1-NRF2 pathway can induce the survival of hepatocytes with excessive DNA damage, and corresponding mutations were found in human HCC samples [45]. Excessive metabolic substrates inhibit the autophagy pathway, helping the hepatocyte with DNA damage escape from autophagy-dependent cell death [46]. Impaired DNA damage repair has been demonstrated in NASH patients. Schults et al. found that NASH patients with high neutrophilic influx had significantly reduced nucleotide excision repair capacity resulting from impaired damage recognition [47]. Non-homologous end-joining (NHEJ) for DNA double-strand break repair has been found to increase in HCC, and the key enzyme DNA-PK is upregulated and associated with poor survival [48]. NHEJ can be error-prone in certain situations, and increased NHEJ can promote HCC initiation and progression. Although the relationship between changes in NHEJ and NAFLD/NASH is not yet established, some hints are present. For example, the critical enzyme in the de novo fatty acid synthesis, FASN, has been found to upregulate NHEJ [49].

#### 3.2.2. Hepatic Inflammation and Dysregulated Immunity

The role of chronic hepatic inflammation and dysregulated immunity of NASH in HCC development has been extensively studied. Its pivotal role has been established where dysregulations promoting hepatocarcinogenesis are found in both the innate and adaptive immune population, including macrophages, T cells, NK (natural killer) cells, B cells, and others.

Hepatic macrophages in the NASH condition can be very heterogeneous. According to their origin and activation status together with the help of novel single-cell sequencing technology, hepatic macrophages in NASH can be subset into tissue-resident Kupffer cells, monocyte-derived temporary macrophages, monocyte-derived inflammatory macrophages, and others [50]. In mechanistic studies, macrophages are usually classified into Kupffer cells and infiltrating macrophage/monocyte-derived macrophages, or functionally categorized into classically activated M1 or alternatively activated M2 macrophages, albeit an oversimplified model. It is not until recently that the detailed transcriptional difference between the Kupffer cell and hepatic infiltrating macrophage is appreciated [51]. Alterations in the hepatic macrophage have been shown as important in driving the pathogenesis and progression of NASH as well as HCC development in NASH. The hepatic macrophage is commonly believed to go through M1 polarization in NAFLD and NASH in response to hepatocyte damages and cytokines or immunogens from the leaky gut, which, in turn, increases pro-inflammatory cytokine and ROS release and further stresses the hepatocyte and augments the inflammation. Pro-inflammatory cytokines also induce insulin resistance by activating NF-κB and JNK pathways [52]. Although there is some new insight about whether the macrophage goes through M1 activation in early NAFLD or obesity rather than influencing hepatic homeostasis with non-inflammatory factors, there is little doubt that, in fully established NASH, hepatic macrophages are more pro-inflammatory. In contrast, while chronic hepatic inflammation is the rising ground for HCC, in the initiation and progression of HCC, hepatic macrophages are observed to undergo M2 activation that induces immunosuppression and produces mitogens to promote survival and proliferation of tumor-initiating cells or tumor cells. It has been demonstrated in the diethylnitrosamine (DEN) induced mouse model of HCC that the western diet can induce NASH and promote macrophage M2 activation as well as HCC development [53]. Myeloid-derived suppressor cells (MDSCs) are a heterogeneous population of myeloid cells whose number increase in pathological conditions, including NASH and HCC [54,55,56]. MDSCs have a potent immunosuppressive effect, and their activities against the anti-tumor function of hepatic macrophage and cytotoxic CD8 T cells have been observed in HCC [57,58].

The cytotoxic CD8 T cell is essential in the clearance of malignant cells, and its number in NASH liver increases in response to pro-inflammatory cytokines. However, an increased number and enhanced activation of CD8 T cells have been suggested to promote NASH establishment and NASH to HCC transition in a mouse model for NASH [59]. Moreover, constant activation of CD8 T cells, which is frequently seen in the NASH condition, will induce CD8 T cell exhaustion and the activation of other immunosuppressive types of machinery, leading to the loss of surveillance against malignant cells eventually. In the pathogenesis and progression of NASH, the CD4 T cell is biased toward Th1 and Th17 phenotypes and promotes steatosis and inflammation [60,61,62]. In contrast, similar to the notion that the tumor-targeting helper CD4 T cell is usually beneficial in the HCC stage, loss of specific subtypes of CD4 T cells that presumably recognize a neoantigen is important in the progression from NASH to HCC. As shown by Ma et al., a significantly decreased CD4 T cell number could be seen in different mouse models for NASH-related HCC in the NAFLD stage, and CD4 T cell depletion caused more hepatic tumor lesions [63]. The reduced CD4 T cell might be due to cell death induced by excessive hepatic fatty acids taken up by CD4 T cells and subsequent increased mitochondrial ROS production [63]. A subset of CD4 T cells, the regulatory T cells (Tregs), are important in restricting inflammation and the resolution of the wound healing process. Thus, Treg can be beneficial in controlling NASH progression and in the recession of fibrosis. A decreased Treg cell number was seen in several studies using mouse models of NAFLD as reviewed by Van Herck et al. [60], which is likely the result of elevated ROS [64]. In contrast, in the HCC stage, Treg is increased in patients and is preferably located at the tumoral lesion [65,66]. Functional assays indicated that HCC-associated Treg could inhibit CD8 T cell expansion [66]. However, no study has provided in-depth insight explaining if the transition from decreased Treg in the NASH stage toward increased Treg in the HCC site is regulated by the tumor cell or is an event that could appear in the NASH condition in response to high CD8 T cell activity and fibrosis/cirrhosis.

The NK cell is the innate cytolytic cell with a tumor surveillance role and is particularly abundant in the liver in both humans and mice [67]. Apart from direct tumor surveillance, NK cells can also induce the apoptosis of hepatic stellate cells, thus, restricting fibrosis [68]. While the NK cell is beneficial against the progression from NASH to HCC in these ways, NK cells can promote hepatic inflammation through cytokine secretion and induction of hepatocyte death. NK cells have been demonstrated to play a malevolent role promoting chronic inflammation of NASH in the mouse model [69,70], and an immunoregulatory role of the NK cell on polarizing macrophages toward M1-like phenotypes has been described in a mouse model of NASH [71]. However, a recent study showed that, in a human cohort, the number, frequency, phenotype, and functionality of NK cells in both the circulation and tissues, including adipose tissue and liver, was not substantially changed in biopsy-confirmed NAFL or NASH patients as compared to healthy controls [72]. While the clinical relevance of NK cell’s role in NASH is still under debate, reduced NK cell number and activity have been reported in human HCC and were associated with poor prognosis [73]. The abnormal NK cells with an inhibited activity in the HCC microenvironment could be induced by several factors, including increased Tregs, increased MDSCs, and high expression of immune checkpoint receptor Tim-3 in the NK cell [73,74,75]. An intriguing question would be whether the chronic inflammation in NASH could exhaust NK cells and contribute to the suppressive phenotype of NK cells in HCC [76], although research showed that the NK cell might not be changed in NASH patients [72]. A recent study shed some light on this question. Nishina et al. demonstrated that CD26 expressed by the HCC cell serves as a peptidase (DPP4) to truncate chemokine CXCL10 and restricts NK cell recruitment and activation. Using a mouse model for NASH-related HCC, they showed that continuous administration of the DPP4 inhibitor to mice started around the time of NASH development did not improve the parameter of NASH, but did reduce liver tumor volume and number and increase the infiltration of activated NK cells to both the tumor and non-tumor liver [77]. Further study is needed to elucidate if the NK cell defects in NASH-related HCC are solely induced by the tumor cell and tumor microenvironment.

The B cell plays an active role in promoting NAFLD/NASH pathogenesis and progression through antibody-dependent and independent mechanisms [78,79,80,81]. On the other hand, high B cell infiltration to HCC correlated with prolonged survival after curative resection, which could result from the anti-tumor immunoglobulin production of B cells [82]. Moreover, Zhang et al. found that, in their HCC patient cohorts whose etiology was not revealed, the density of B cells decreased in tumors when compared to non-tumor livers, and increased B cell infiltration to the tumor was associated with better survival [83]. Similar to other immune cells, not all subtypes of B cells are friendly in HCC. A PD-1^hi^ B cell subset has been identified in human HCC, which can suppress anti-tumoral T cell immunity and promote tumor growth through IL-10 secretion [84]. B cells with an FcγRII^low/−^ activated phenotype have been found in human HCC with a high frequency, which also suppresses cytotoxic T cell activity [85]. The generation of pro-tumor immunosuppressive B cells has been linked to NASH. The accumulation of IgA-producing cells that also express PD-L1 and IL-10 and suppress cytotoxic CD8 T cells has been demonstrated in humans and mice with NAFLD [86]. The generation of immunosuppressive B cells could correspond to the need to limit CD8 T cell-induced tissue damage in NASH conditions and induced by factors produced by hepatic macrophages and CD4 T cells. Thus, B cells, depending on their phenotypes, play different roles in both the NASH and HCC stages, where immunosuppressive B cells are generated to restrict chronic inflammation of NASH fueled by diverse immune populations, including B cells. However, this type of B cell also compromises CD8 T cell activity, resulting in HCC initiation.

Taken together, the dysregulated immunities in NASH and HCC have many distinctions, which are also reviewed elsewhere [87]. Yet, the transitions from the dysregulated immunity of NASH to a differently dysregulated immunity in HCC could rationally occur, altering several types of immune cells. For example, the chronic activation of CD8 T cells in NASH induces tissue damage and NASH progression, and, at the same time, activates immunosuppressive types of machinery and promotes T cell exhaustion, leading to reduced activity of CD8 T cells, surveillance evasion of tumor-initiating cells, and, subsequently, enhanced immunosuppression driven by cancer cells. As shown in many studies using mouse models, attenuation of dysregulated immunities either in the NASH stage or the HCC stage could inhibit the initiation and progression of NASH-related HCC.

#### 3.2.3. Cirrhosis

While NASH-related HCC can develop in the absence of cirrhosis or fibrosis, cirrhosis is associated with a higher risk of HCC in NASH conditions, as demonstrated in many observational studies, including a recent one. A retrospective cohort study in the US matching 296,707 NAFLD patients with controls found an annual incidence of 10.6/1000 person-years in NAFLD patients with cirrhosis versus 0.08/1000 person-years in those without cirrhosis [88]. Fibrosis, which is the key feature of cirrhosis, is induced by chronic necroinflammation in NASH. In this process, the hepatic stellate cells are activated and trans-differentiated into the myofibroblasts that produce the extracellular matrix (ECM). ECM is usually made to maintain the liver structure and can be gradually removed after acute or curable liver injury, as the reproduction of hepatocytes and proceedings of wound healing. However, in a chronic injury like NASH, excessive ECM persists and accumulates, leading to cirrhosis and permanent loss of liver function. Elastography can be used to non-invasively monitor the development of liver cirrhosis in NASH patients when factoring skin-to-liver distance into the data interpretation [89]. By evaluating the association between liver stiffness measured by elastography and seven commonly employed biomarkers for the diagnosis of liver fibrosis, a recent study found that those biomarkers are doubtful in determining liver stiffness in the general population [90]. An important question would be: is cirrhosis that more frequently than not precedes NASH-related HCC a direct contributor to HCC development or only an indicator for the severity of necroinflammation in NASH and a part of the constant tissue regeneration process? The opposite view of the common notion that cirrhosis is a risk factor for HCC is continuously rising, suggesting that fibrosis may impose physical space restriction on HCC development and promote immunosurveillance, thus, impeding HCC development [91]. Considering this view and the fact that most NASH patients do not have cirrhosis, and NASH-related HCC can develop in the absence of cirrhosis [88], prevention and treatment methods for NASH-related HCC should not be limited to those that possess the effect to improve cirrhosis. This is especially reasonable for the treatment of cancer since it has been shown in pancreatic ductal adenocarcinoma (PDAC) patients that fewer myofibroblasts in the tumor correlated with reduced survival, and in mouse models for PDAC, the depletion of myofibroblast and the reduction of tumor-stromal content has a detrimental rather than a beneficial effect [92,93]. Nevertheless, since NASH might progress to liver cirrhosis and HCC, monitoring liver stiffness by non-invasive elastography might help identify medication to inhibit cirrhosis progression and reduce the risk for HCC in NASH patients.

## 4. Direct Effects and Underlying Mechanisms of Metformin on Hepatocytes or Malignant Cells That May Inhibit NASH-Related HCC

### 4.1. Metformin and Substrate Overload in Hepatocyte

**Figure 2:**
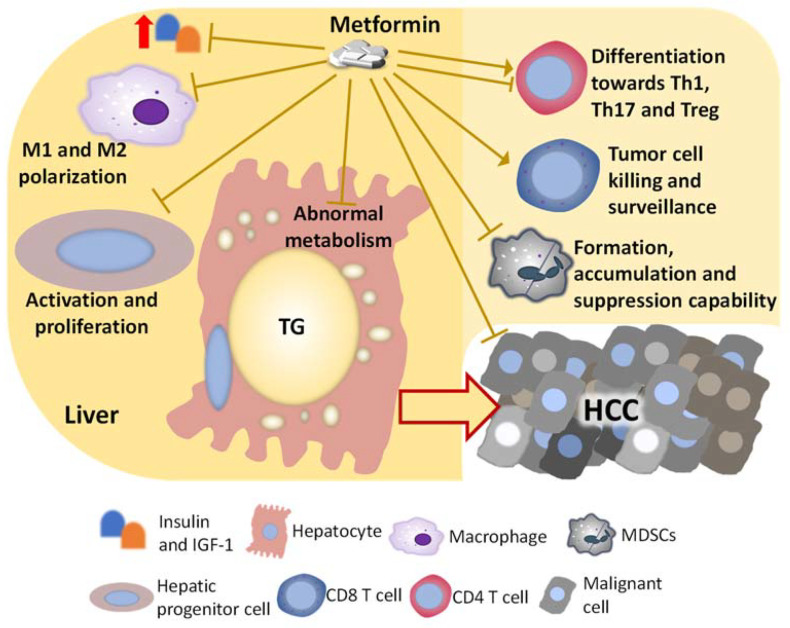
Factors and cells changed by metformin, which mediates the liver-protecting effect against HCC development in the NASH condition. The mechanism by which metformin inhibits the development of NASH-related HCC is multi-factorial. Metformin directly impacts on hepatocytes, hepatic progenitor cells, and HCC cells, which suppress malignant transformation and cancer progression. Besides influencing those cells that go through the malignant transformation directly, metformin also changes the activity and population of immune cells including macrophages, T cells, and MDSCs, suppressing the HCC development.

Due to its glucose-lowering effect, metformin is likely to relieve the hepatocyte from substrate overload to some extent, which is the stem of NASH pathogenesis, and is associated with the generation of oxidative stress vital in NASH-related HCC development (Figure 2). The glucose-lowering effect of metformin is primarily attributed to its inhibition of hepatocyte gluconeogenesis. However, the underlying mechanism that once seemed to be coined as mediated by the direct inhibition of mitochondrial respiratory complex I by metformin, is still under active debate [94,95]. Despite controversies, metformin is thought to be enriched to mitochondria through the attraction imposed by the mitochondrial membrane potential or through protein-mediated transportation, resulting in a high concentration of the drug and inhibits the mitochondrial respiratory chain, which decreases the ATP/AMP ratio. This altered energy balance in hepatocytes is thought to be the key factor that decreases hepatic gluconeogenesis through multiple pathways. These pathways involve AMP-activated protein kinase (AMPK), fructose 1,6-bisphosphatase (FBP1), adenylate cyclase (AC), acetyl CoA carboxylase (ACC), and others, with many among them having an additional role in NASH pathogenesis and hepatocarcinogenesis. Before discussing these players, it is worth noting that the inhibition of gluconeogenesis and mitochondrial respiratory complex I could be beneficial to NASH-related HCC in several ways. First, inhibition of gluconeogenesis could improve the substrate overload in the hepatocyte, reducing cell stress and postponing the progression of T2DM and NAFLD. It also helps with lowering oncogenic insulin and IGF-1 levels. Inhibition of mitochondrial respiratory complex I by metformin in cancer cells has also been demonstrated, which leads to reduced proliferation, albeit some cancer cell lines are less sensitive due to low dependency to mitochondrial respiration [96,97,98].

### 4.2. Metformin and AMPK Signaling

Of those factors regulated by an altered cell energy state induced by metformin, the protein kinase AMPK is highly relevant to NASH-related HCC. Metformin treatment is generally believed to activate AMPK by lowering the cell energy state that promotes the phosphorylation of AMPK by LKB1, even though alternative mechanisms were also proposed [99]. Activated AMPK phosphorylates and inactivates ACCs (ACC1 and ACC2), which are the key enzymes of de novo fatty acid synthesis and the enzyme that produces inhibitors to the fatty acid β-oxidation, respectively [100,101,102]. Thus, the activation of AMPK by metformin can alleviate the substrate overload in hepatocytes by reducing de novo fatty acid synthesis and promoting fatty acid β-oxidation, which could postpone NASH development and progression. A recent study combined RNA-seq and ChIP-seq technology in the primary human hepatocyte culture system and discovered novel genes regulated by metformin treatment in an AMPK-dependent manner. Some of these novel genes, including *KLF6* and *ATF3*, play roles in regulating lipid and glucose metabolism in the hepatocyte [103]. Moreover, AMPK signaling has been highlighted in hepatocarcinogenesis and HCC progression, as shown by clinical observations that cirrhosis patients with a low level of AMPK activating phosphorylation having a higher risk for HCC [104], and a low level of AMPK activating phosphorylation in HCC patients with HBV etiology correlating with metastasis and a poor prognosis [11]. In the latter study, metformin successfully induced AMPK activation in cultured human HCC cells and inhibited cell growth [11]. Metformin has been shown to suppress the hepatocarcinogenesis of a steatosis-associated mouse liver tumor model of oncogene AKT/c-Met overexpression, where AMPK activation by metformin treatment was seen. Activation of AMPK in cultured human HCC cells by metformin was also shown by the same study [105]. Since the anti-HCC effect and underlying mechanisms of AMPK activation are not specific to NASH-related HCC and have been recently reviewed, this topic will not be discussed here in detail. Still, the mechanisms are generally related to the role of AMPK as a sensor for energy deprivation, where AMPK activation halts the cell cycle and inhibits cell anabolism. The anti-cancer effect of metformin has been attributed to AMPK activation [106]. It is unknown whether NASH-related HCC has more potent or more frequent AMPK inactivation. In the NAFLD/NASH stage, AMPK inhibition in substrate-overloaded hepatocytes is conceivable and commonly reported [107,108]. However, some questions are not addressed yet. For example, do the cancer cells that are transformed from hepatocytes with constant AMPK inhibition keep this trait? Furthermore, do cancer cells in the NASH environment adopt more potent AMPK inhibition that renders them more sensitive to metformin?

### 4.3. Metformin and Other Factors Regulated by AMP

Metformin has also been shown to inhibit gluconeogenesis by lowering the energy state independent of AMPK activation but by inhibiting FBP1 or AC with increased AMP [109,110]. FBP1 and the product of AC, cAMP, have their role in HCC development. However, inhibition of FBP1 or lowering the cAMP level was shown to be detrimental rather than beneficial in this scenario [111,112,113]. More relevant to NASH-related HCC is that the loss of FBP1 expression induced mild NAFLD-like features in mice and accelerated the progression of the carcinogen-induced liver tumor [112]. In this aspect, using metformin to treat HCC might add fuel to the fire. Further studies that examine these activities of metformin in cancer cells are needed.

### 4.4. Metformin and Oxidative Stress

Metformin has been demonstrated to alternatively regulate gluconeogenesis by regulating the hepatocyte redox state. It promotes a more reduced cytosol, albeit this effect might be the by-products of the inhibition of the mitochondrial respiratory complex I [94]. The altered hepatocyte redox state by metformin might alleviate oxidative stress, preventing NAFLD progression and hepatocarcinogenesis. Metformin was shown to inhibit oxidative stress-induced apoptosis in primary rat hepatocytes. However, this study did not indicate if metformin reduced the amount of oxidative product generated by ROS-inducing drugs but attributed the protective effect of metformin to its JNK inhibition and HO-1 induction [114]. Metformin was also shown to promote the activation of NRF2, the master regulator of antioxidative response, in the liver of animal models for T2DM or hepatotoxicity [115,116]. AMPK can activate NRF2 by directly phosphorylating NRF2 or inhibiting GSK3β, which is an inhibitory regulator of NRF2 [117]. In addition, metformin has been shown to activate NRF2 and attenuate oxidative stress independent of AMPK in mouse primary brain endothelial cells [118]. However, the activity of metformin on NRF2 in malignant cells contradicts those shown in non-malignant cells. NRF2 activation in malignant cells helps them to gain survival advantages and mediates drug resistance [119]. Instead of activating NRF2, metformin was shown to induce the downregulation or inactivation of NRF2 in cancer cells. Do et al. showed that metformin reduced Nrf2 mRNA by inhibiting the Raf-ERK pathway independent of the AMPK pathway in the human HCC cell HepG2 [120]. When treating non-small cell lung cancer (NSCLC) cells with a potential chemo-preventive agent, Yu et al. found that NRF2 signaling was activated, and it can be inhibited by metformin co-treatment. In this study, metformin treatment alone was found to promote NRF2 S40 phosphorylation, which is seen in NRF2 activation, but metformin treatment also decreased the acetylation of NRF2 and reduced its activity [121]. It was recently found that metformin augmented the cytotoxic effect of cisplatin to NSCLC cells by promoting NRF2 degradation through a mechanism similar to that of HepG2 cells, which is done by inhibiting Raf-ERK [122]. When treating lung adenocarcinoma cells with metformin, Zhang et al. found that NRF2 expression was also reduced through ERK1/2 and PI3K-AKT pathway inhibition by metformin [123]. Isocitrate dehydrogenase 1- (IDH1) mediated chemoresistance in endometrial cancer cells can be abated by metformin treatment, which inhibits the IDH1-induced NRF2 expression [124]. Metformin seems to play opposite roles in terms of NRF2 activation in the malignant and non-malignant cells, which could be owing to different upstream pathways activating NRF2 in the malignant cell as compared to the non-malignant cell, or due to the fact that most studies testing metformin on malignant cells were done in the cell culture system. Despite different effects, current data support that metformin treatment is beneficial in both the NASH and HCC stages in regulating oxidative stress and NRF2 activation.

### 4.5. Metformin and Hepatic Progenitor Cells

Hepatic progenitor cells (HPCs) are bipotential stem cells that can differentiate into both hepatocyte and cholangiocyte. During chronic liver diseases, including NASH, HPCs are activated and increased to facilitate liver regeneration apart from hepatocytes’ replication [125]. There are different theories, but HPCs can be the origin of HCC cells, and even if HPCs do not directly become cancer cells, they are generally believed to promote carcinogenesis [126]. HPC activation and differentiation are induced by hepatocyte damage and are supported by activated hepatic stellate cells and hepatic macrophages during chronic liver diseases. Thus, hepatocyte-protecting metformin may reduce the number and activation of HPCs to reduce HCC risk in NASH conditions. In a rat model of cirrhosis, metformin was found to reduce HCC incidence by inhibiting HPC activation [127]. However, the underlying mechanism was not elucidated in this study. It is unclear which type of cells metformin acts on to inhibit HPC activation, whether this effect is directly on HPC, indirectly on the immune population, or remotely on reducing hepatocyte cell death. A more relevant study was conducted on a unique *Ncoa5* deletion mouse model for T2DM and NASH accompanied HCC. Haploid *Ncoa5* deficiency induced the appearance of T2DM and NASH features in mice fed a standard diet and caused spontaneous development of HCC, which can be partially attributed to a high expression of pro-inflammatory cytokine IL-6 [128]. In the following study, the group further characterized the oncogenic liver environment and found increased HPC number concurrent with high expression of p21 (p21^WAF1/CIP1^) in hepatocytes. Metformin was shown to reduce the HCC incidence in this mouse model while reducing p21 expression in hepatocytes and decreasing the HPC number. Deletion of the *p21* gene phenocopied metformin treatment in *Ncoa5* deficient mice with regard to the reduced HPC number [129]. Thus, metformin may reduce HCC risk in the NASH condition partially by inhibiting HPC activation by reducing p21 expression in hepatocytes. Although metformin has been shown to inhibit p21 expression through AMPK [130], and increased expression of p21 in hepatocytes has been found to increase HPC number 25 years ago [131], it is still not clear how high expression of p21 in hepatocytes promotes HPC activation.

## 5. Metformin on the Immune Population That May Indirectly Inhibit NASH-Related HCC Development

As discussed previously, the immunity in the NASH liver is dysregulated and is generally pro-inflammatory, which stresses and damages the hepatocyte, promoting the accumulation of genetic and epigenetic alterations. Several immunosuppressive components also exist in the dysregulated immunity in the NASH liver, such as M2 macrophages, MDSCs, immunosuppressive B cells, exhausted CD8 T cells, and Tregs, and these components permit the survival and growth of tumor-initiating cells. Metformin has been frequently shown to improve the dysregulated immunity in the liver with chronic diseases including NASH and HCC, which could be partially attributed to the direct hepatocyte-protecting effect, but metformin is also shown to directly act on immune cells (Figure 2). In this part, we will discuss the impact of metformin on immune cells that indirectly inhibit the transformation of hepatocytes in the NASH condition and suppress the progression of NASH-related HCC.

### 5.1. Metformin on Macrophages

Suppression of the macrophage activation toward the M1 or M2 phenotype depending on the microenvironment of the specific disease stages could be beneficial to NASH and NASH-related HCC. Inhibiting the M1-related pro-inflammatory activity of macrophages in the early stage of NASH could improve insulin sensitivity [132] and reduce the stress to hepatocytes. At the same time, such an inhibition in the tumor or tumor-initiating-cell-bearing liver could be detrimental. Inhibiting the M2-related immuno-modulatory activity of macrophages can remove the permit and support for cancer cell outgrowth.

In the *Ncoa5* deletion mouse model for NASH-related HCC, pro-inflammatory cytokines, including IL-6 produced by the hepatic macrophage, were increased before the onset of HCC, which is concurrent with an increased intrahepatic macrophage number. The M2 macrophage percentage and number in the pre-HCC stage were also increased. This finding suggested a unique hepatic microenvironment of NCOA5-deficient mice that disturbs the hepatocyte and facilitates tumorigenesis simultaneously in terms of macrophage function. Long-term metformin treatment decreased the total intrahepatic myeloid cell number and the M2 macrophage incidence in *Ncoa5*^+/−^ mice. Metformin seems to regulate both the M1 and M2 population in the NASH condition here, and the mechanism was elusive but was implied to metformin’s repression of p21 expression in the hepatocyte [129]. In a transgenic zebrafish model of HCC driven by the expression of activated β-catenin, a high-fat diet was found to promote HCC progression. Treating the NASH-related HCC of this β-catenin/high-fat diet model with metformin reverted the accelerated progression, but HCC persisted. In this process, the elevated M1-polarization of macrophages induced by a high-fat diet was reduced by metformin [133]. Mechanistically, how the decreased M1 macrophage characterized by TNFα expression inhibits NASH-related HCC progression was not addressed in this study. It might be related to the resistance to TNFα-induced cell death of tumor cells bearing a transgenic oncogene and, contrastingly, the augmented effect of TNFα in killing cytotoxic T cells and modulating MDSCs and Tregs [134]. The direct inhibition of metformin to M1 activation induced by lipopolysaccharides (LPS) has been demonstrated in vitro using human monocytes and mouse macrophages [135,136]. Mechanistically, these two studies both showed that metformin inhibited MAPK activation but also conflicted with each other, as one stated that metformin also inhibited NF-κB activation while the other said the opposite. Kim et al. demonstrated that the MAPK inhibition by metformin in mouse macrophages was AMPK-dependent, while whether the same is true for NF-κB inhibition is still under debate [136]. Using the human monocytic leukemia (THP-1) cell line, Vasamsetti et al. showed that metformin could inhibit monocyte-to-macrophage differentiation via AMPK activation [137]. A similar concept could be significant in NASH-related HCC because the monocyte-derived macrophage is currently regarded as an important source of the pro-inflammatory macrophage in the NASH liver.

Inhibition of macrophage M2 polarization by metformin was also reported. In the cell culture system, metformin was shown to suppress the M2 polarization of macrophage-like murine RAW264.7 cells stimulated with IL-13. The inhibitory effect is related to AMPK activation [138]. A similar result was seen in IL-4-stimulated RAW264.7 cells, murine bone marrow-derived macrophages, THP-1-originated macrophages, and human peripheral blood monocyte-derived macrophages, where mannose-modified, macrophage-derived microparticles loaded with metformin converted the M2 phenotype toward M1. In this study, such microparticles loaded with metformin were also effective in an animal model to reprogram the tumor immune microenvironment [139]. In addition, metformin was reported to act on the cancer cell to reduce the M2 polarization of the tumor-associated macrophage. The study was done by treating breast cancer cells with metformin and then using a medium conditioned by cancer cells to treat THP-1 cells. Cancer cells treated with metformin were found to produce less M2-inducing cytokines and more M1-inducing cytokines, and, again, this was shown to relate to AMPK inhibition in the cancer cell [140]. Altogether, metformin might inhibit both M1 and M2 phenotypes of macrophages and inhibit the hepatic seeding of macrophages derived from the monocyte in the NASH liver, as shown by Williams et al. Moreover, metformin could inhibit M2 polarization of the tumor-associated macrophage in the HCC stage, reinforcing the benefit of metformin use in NASH-related HCC.

### 5.2. Metformin on MDSCs

Inhibiting the formation and recruitment of MDSCs in the liver would alleviate the immunosuppressive microenvironment that promotes tumor initiation and progression. The accumulation of MDSCs in the NASH liver of *Ncoa5*^+/−^ mice can be prevented by long-term treatment of metformin, concurrent with a reduced HCC incidence [129]. The reduction of hepatic MDSCs could be the result of suppressed chronic inflammation in the liver by metformin. In the tumor-microenvironment, metformin might also suppress MDSCs’ accumulation. In patients with esophageal squamous cell carcinoma (ESCC), those with diabetes and treated with metformin had significantly less MDSC infiltration in the tumor as compared to those with or without diabetes that were not treated with metformin [141]. The effect was mediated by AMPK activation and subsequent NF-κB inhibition in ESCC cells, and reduced production of chemoattractants for MDSCs. More directly, it has been recently reviewed that the AMPK pathway plays a potential role in regulating MDSC functions, where metformin may inhibit the immunosuppressive function of MDSCs [142]. Metformin treatment reduced the expression and activity of CD39 and CD73 related to immunosuppression in MDSCs isolated from ovarian cancer patients through AMPK activation and subsequent HIF-1α suppression in MDSCs. The group also showed that metformin treatment in ovarian cancer patients with diabetes decreased CD39 and CD73 in their MDSCs and increased effective CD8 T cell percentage, which is concurrent with prolonged survival [143]. The AMPK-related inhibition of MDSC functions by metformin was also observed in a syngeneic tumor mouse model [144]. Investigation examining the inhibition of MDSC accumulation and function by metformin in HCC is still lacking, but the discovery of such inhibition in other cancers may apply to the pro-tumorigenic NASH microenvironment and the tumor microenvironment of NASH-related HCC.

### 5.3. Metformin on T Cells

Inhibiting CD8 T cell infiltration and activation in the early NASH stage can alleviate hepatocyte damage and prevent the activation of immunomodulatory machinery and T cell exhaustion. However, the immunosurveillance by CD8 T cells could be dampened during such an inhibition. In contrast, boosting CD8 T cell expansion and cytotoxicity in the HCC stage can help with tumor clearance. In the NASH liver of the NCOA5-deficient mouse, the increase of hepatic CD8 T cells, which was more likely associated with activated and tissue-resident memory phenotypes, was prevented by long-term metformin treatment concurrent with a reduced enrichment of T cell exhaustion gene signatures in the liver transcriptome [129]. The authors suggested a mechanism related to metformin’s suppression to chronic hepatic inflammation that caused the observed reduction of T cell infiltration and exhaustion. While the study above showed the likely indirect effect of metformin on CD8 T cells in pre-HCC NASH liver, a direct effect of metformin on CD8 T cells was also demonstrated. Murine CD8 T cells treated with metformin more strongly inhibited the tumor growth of a melanoma intradermal inoculation mouse model than non-treated CD8 T cells, and the change depends on AMPK activation by metformin. The same study also showed that oral administration of metformin increased the tumor-infiltration of CD8 T cells and protected them from apoptosis and exhaustion in tumor inoculation mouse models [145]. A similar effect was also seen in the human cell. Treating human peripheral and tumor-infiltrating CD8 T cells with metformin in vitro increased the frequency of central memory and memory stem subtypes with increased expression of the proliferation marker and elevated resistance to apoptosis. The expressions of immune checkpoint molecules PD-1 and Tim-3 decreased, whereas the activation marker CD69 increased in CD8 T cells treated with metformin. HER2 CAR (chimeric antigen receptor) T cells treated with metformin also had a more potent cytotoxic activity. The mechanism was predicted to be related to AMPK activation by metformin. Notably, the human CD8 T cell used in this study was primarily from lung cancer patients with diabetes, making it relevant for NASH-related HCC [146]. PD-L1 has recently been found to be phosphorylated by metformin-activated AMPK, and, subsequently, went through ER accumulation and ER-associated protein degradation, leading to enhanced cytotoxicity of T cells [147]. Altogether, it is commonly reported that metformin can boost the anti-tumor activity of CD8 T cells, supporting the use of metformin in NASH-related HCC.

Studies indicated that metformin also regulates the function of CD4 T cells. Direct inhibition of CD4 T cell’s production of IL-22, an HCC-promoting cytokine [148], by metformin has been demonstrated. The effect is mediated by inhibiting the differentiation of CD4 T cells toward Th1 and Th17 cells, possibly via AMPK activation by metformin. In the orthotopic mouse model of HCC, metformin treatment reduced the elevated IL-22 level in the serum of tumor-bearing mice and decreased the splenic Th1 and Th17 cell numbers [149]. The differentiation toward and the function of Treg can be regulated by metformin. In the cell culture system, metformin pre-treatment inhibited the TGF-β-induced CD4 T cell differentiation toward Treg. The resulting Treg with metformin pre-treatment had impaired ability to suppress CD8 T cells. The effect was shown to involve AMPK and mTOR since inhibiting either factor reversed metformin’s suppression to cell differentiation toward Treg. However, this is against the common notion that mTOR is targeted for inactivation by AMPK activation. Albeit the unclear mechanism, reduced Treg tumor-infiltration and Treg function by metformin treatment were also found in the intradermal inoculation mouse model of cancer in the same study [150]. It should be noted that, although such an inhibition of Treg by metformin can be beneficial in the HCC stage, the immunoregulatory Treg is important in protecting the liver in the NASH stage. In studies related to autoimmune diseases, metformin was shown to promote rather than inhibiting the differentiation toward Treg [151,152]. Metformin may play distinct roles in the differentiation process toward Treg induced by various factors. Still, a consensus, to some extent, has been reached by these studies on metformin’s ability to suppress the differentiation of CD4 T cells toward Th1 and Th17 cells.

## 6. Conclusions

The prevalence of NAFLD and the rising incidence of NASH-related HCC urge further investigations to understand the mechanism of HCC development with this etiology and find effective drugs to prevent and treat NASH-related HCC. It is supported by accumulating data that metformin can ameliorate NAFLD/NASH-inducing conditions and improve the HCC-inducing features of NASH. Metformin has been shown to act on hepatocytes, HCC cells, and various immune populations to suppress HCC development. Metformin has been recently highlighted in modulating the immunity of the liver against HCC development, and our knowledge is still empty in some areas of this topic. For example, studies examining the effect of metformin on B cells and NK cells in NASH and HCC are still lacking. Clinical studies examining the impact of metformin use in NAFLD/NASH patients on HCC incidence and prognosis start to emerge but are still under-investigated. Hopefully, this review can inspire additional studies toward elucidating metformin’s mechanisms of action against NASH-related HCC development on hepatocytes and the immune population as well as provide rationales for the clinical evaluation of metformin in preventing and treating NASH-related HCC.

## Data Availability

Not applicable.
